# Towards an early warning system for monitoring of cancer patients using hybrid interactive machine learning

**DOI:** 10.3389/fdgth.2024.1443987

**Published:** 2024-08-14

**Authors:** Andreas Trojan, Emanuele Laurenzi, Stephan Jüngling, Sven Roth, Michael Kiessling, Ziad Atassi, Yannick Kadvany, Meinrad Mannhart, Christian Jackisch, Gerd Kullak-Ublick, Hans Friedrich Witschel

**Affiliations:** ^1^ Oncology, Breast Center Zürichsee, Horgen, Switzerland; ^2^ Clinic for Clinical Pharmacology and Toxicology, University Hospital, Zürich, Switzerland; ^3^ FHNW, University of Applied Sciences and Arts Northwestern Switzerland, Olten, Switzerland; ^4^ Mobile Health AG, Zürich, Switzerland; ^5^ Onko-Hämatologisches Zentrum Zug, Zug, Switzerland; ^6^ Sana Klinikum Offenbach GmbH, Offenbach, Germany

**Keywords:** cancer, ePROs, systemic therapy, digital patient monitoring, interactive machine learning, early warning systems (EWS)

## Abstract

**Background:**

The use of smartphone apps in cancer patients undergoing systemic treatment can promote the early detection of symptoms and therapy side effects and may be supported by machine learning (ML) for timely adaptation of therapies and reduction of adverse events and unplanned admissions.

**Objective:**

We aimed to create an Early Warning System (EWS) to predict situations where supportive interventions become necessary to prevent unplanned visits. For this, dynamically collected standardized electronic patient reported outcome (ePRO) data were analyzed in context with the patient's individual journey. Information on well-being, vital parameters, medication, and free text were also considered for establishing a hybrid ML model. The goal was to integrate both the strengths of ML in sifting through large amounts of data and the long-standing experience of human experts. Given the limitations of highly imbalanced datasets (where only very few adverse events are present) and the limitations of humans in overseeing all possible cause of such events, we hypothesize that it should be possible to combine both in order to partially overcome these limitations.

**Methods:**

The prediction of unplanned visits was achieved by employing a white-box ML algorithm (i.e., rule learner), which learned rules from patient data (i.e., ePROs, vital parameters, free text) that were captured via a medical device smartphone app. Those rules indicated situations where patients experienced unplanned visits and, hence, were captured as alert triggers in the EWS. Each rule was evaluated based on a cost matrix, where false negatives (FNs) have higher costs than false positives (FPs, i.e., false alarms). Rules were then ranked according to the costs and priority was given to the least expensive ones. Finally, the rules with higher priority were reviewed by two oncological experts for plausibility check and for extending them with additional conditions. This hybrid approach comprised the application of a sensitive ML algorithm producing several potentially unreliable, but fully human-interpretable and -modifiable rules, which could then be adjusted by human experts.

**Results:**

From a cohort of 214 patients and more than 16’000 available data entries, the machine-learned rule set achieved a recall of 19% on the entire dataset and a precision of 5%. We compared this performance to a set of conditions that a human expert had defined to predict adverse events. This “human baseline” did not discover any of the adverse events recorded in our dataset, i.e., it came with a recall and precision of 0%. Despite more plentiful results were expected by our machine learning approach, the involved medical experts a) had understood and were able to make sense of the rules and b) felt capable to suggest modification to the rules, some of which could potentially increase their precision. Suggested modifications of rules included e.g., adding or tightening certain conditions to make them less sensitive or changing the rule consequences: sometimes further monitoring the situation, applying certain test (such as a CRP test) or applying some simple pain-relieving measures was deemed sufficient, making a costly consultation with the physician unnecessary. We can thus conclude that it is possible to apply machine learning as an inspirational tool that can help human experts to formulate rules for an EWS. While humans seem to lack the ability to define such rules without such support, they are capable of modifying the rules to increase their precision and generalizability.

**Conclusions:**

Learning rules from dynamic ePRO datasets may be used to assist human experts in establishing an early warning system for cancer patients in outpatient settings.

## Introduction

Potential complications in cancer patients undergoing systemic treatments need to be recognized during hospital stays and, in out-patient settings. Continuous monitoring of vital parameters in conjunction with electronic health records provides vast amounts of data which can be used to explore indicative patterns and correlations for predicting patient outcomes (1).The prediction of such outcomes is a progressively increasing field of applications of machine learning in medicine. Current approaches aim at gaining insight into the practice patterns of physicians and at improving physician workforce- forecasting models (2, 3).

Early warning score (EWS) systems are developed to indicate deterioration of common vital parameters such as heart rate, respiration rate, systolic blood pressure, oxygen saturation, and temperature and explored in a variety of clinical and oncological settings (4, 5). Numerous studies have focused on automating medical event and condition predictions such as septic shock, cardiac arrest and hospital re-admission (6–8). Machine learning and artificial intelligence techniques are currently developed for clinical data modeling in pediatric critical care and promoted development of clinical decision support systems (CDSS) (9). In cancer patients undergoing immunotherapies and systemic anticancer treatments a risk factor structure is frequently assessed by big data primarily derived from electronic health records. However, several approaches also use datasets consisting of electronic symptom questionnaires for pre-defined immunotherapy related adverse events (irAEs) (10). Although patient-reported outcomes were recorded electronically as an input for ML and predictive algorithms, this was often not offered in a dynamic manner and potentially harbored a bias towards a focus on medication-specific symptoms.

Earlier, we had developed and implemented an interactive medical device application, medidux^TM^, that enables patients to spontaneously report on more than 90 clinical relevant symptoms as well as wellbeing in a standardized and structured manner (11, 12). The application offers the dynamic electronic reporting of symptoms and their associated grade as defined according to the Common Terminology Criteria of Adverse Events (CTCAE). In addition, the patient's functional status, vital parameters, cognitive state, medication, and free text notes are made available in a validated quality and used in routine clinical practice during chemotherapy and immunotherapeutic interventions. Based on their electronic input, the software notifies patients to contact the treatment team if symptoms are outside the acceptable range. The ensuing patient empowerment is stimulated also through symptom-specific tips offered for the self-management of side effects, may stabilize daily functional activity, and improve communication with treatment teams and timely symptom control thereof (10–12). To date, only a few applications have gained attention and quality with respect to improving efficacy and safety data in clinical trials in oncology. The continuous measurement of ePRO creates a high level of concordance (*κ* = 0.68) for symptom ratings between the patient and treating physician and reliable information for appropriate symptom management (13). Improved assessment, graphical displays for structured reporting on well-being and symptoms, automatic algorithms for alert notifications if symptoms worsen and augmented stakeholder communication allows for early and effective responsiveness and can reduce unplanned visits and hospitalizations in real-world care (12–15).

However, the current integration of ePROs for symptom monitoring and decision making during routine cancer care frequently requires intensive patient-to-physician or nurse specialist communication (15, 16).

Machine learning is understood as an emerging artificial intelligence method, which is widely used to explore predictive factors and establish associated models in healthcare (7, 8) and has been identified as the most successful approach among various data analysis-based methods in clinical risk prediction (17). Although ML has shown success in enhancing predictions, many clinicians may not find such generated algorithms for the purpose an EWS based on out-patient information plausible and helpful (6), often due to a lack of understanding of the reasons for raised alerts. However, future models will (18), emphasize the importance of genuinely human-interpretable models to achieve transparency. Furthermore, interpretable models can become competitive with deep learning, especially when meaningful structured features are present, and their transparency can lead to better models through improved insights during testing. Thus, ML models tend to benefit from incorporating expert knowledge, such as feature weighting, and through the integration of human expertise (19, 20). This approach involves examining the differences between human and ML assessments of clinical risk to derive continuous improvements in the model.

In cancer, only limited data exist on how to stimulate the processes of reviewing patient-reported symptoms to provide algorithms for real-time monitoring and self-care interventions and prevention of unplanned visits and acute admissions (21). To date, “big-data initiatives” may not have adequately included standardized and structured ePROs according to the CTCAE and/or do not provide dynamic real-time analysis. As such, our intention was to narrow this gap by using validated ePRO data from previously published clinical trials (ClinicalTrials.gov NCT03578731) for the development of a real-time EWS for cancer patients, which is based on ML algorithms, but also incorporates expert knowledge. As we targeted patients who were undergoing various anti-cancer treatments (including cytotoxic compounds, antibody-drug conjugates, and checkpoint inhibitors) that often come with significant side effects, as well as other conditions, e.g., infections, we initially focused on identifying and predicting clinical situations that would require immediate medical attention.

By connecting the event of an unplanned visit or hospitalization to the associated reports of symptoms, well-being, vital parameters, medication, and free text, we intended to analyze and learn under what conditions the frequency, quality and grade of data entries and their various combinations might indicate critical situations that will trigger these events. For this, we directly employed human-interpretable models which are easy to understand by physicians and can also be modified accordingly if indicated.

## Material and methods

### Smartphone application

The smartphone app medidux^TM^ was developed to dynamically record symptoms and treatment side effects in cancer patients according to the CTCAE (11, 12) but was not designed to send questionnaires to patients.

Data entry displays for patients allowed the intuitive recording of symptoms, well-being, and activities of daily living, and was implemented with the help of doctors, nurses, and patients. Graphical displays for indicating symptom severity, with easily understandable descriptions based on the CTCAE, could be selected via a horizontal slider (range from 0 to 10). Symptom entries and ratings could be reviewed collaboratively by physicians and patients, and a reliable level of congruency (Қ = 0.68 for common symptoms) between patient- and clinician-reported toxicity in cancer patients receiving systemic therapy has recently been demonstrated for the smartphone app (12). The symptom history was displayed on a timeline with individual colors for each symptom. In addition, diary entries and information on diagnosis and therapy were indicated separately.

Usability and adherence for entering well-being, symptoms and corresponding grading, medications, as well as the “graphical timeline” of the patients’ history of symptoms have been described in previous publications (11, 12).

Patients could also add private notes or additional symptoms and any medical measures undertaken as free text or in a structured manner, and daily functional activity according to the Eastern Cooperative Oncology Group (ECOG) performance status and reviewed information for self-care (symptom specific tips) were displayed by the app depending on the severity of symptoms upon data entry. Structured vital parameter acquisition was available for body temperature, blood pressure, oxygen saturation, weight, and blood glucose level. The history of recorded data was displayed automatically also to care teams.

### Patient demographics

Patient data for analysis were derived from a retrospective multicenter, observational study that had collected ePROs from 214 patients through a certified medical device application (NCT03578731). Results from the study demonstrated a substantial congruency (Қ = 0.68) for common symptoms of collaborative symptom grading between patients and physicians, indicating a high data quality made available for the hybrid ML approach. (11, 12).

**Figure 1:**
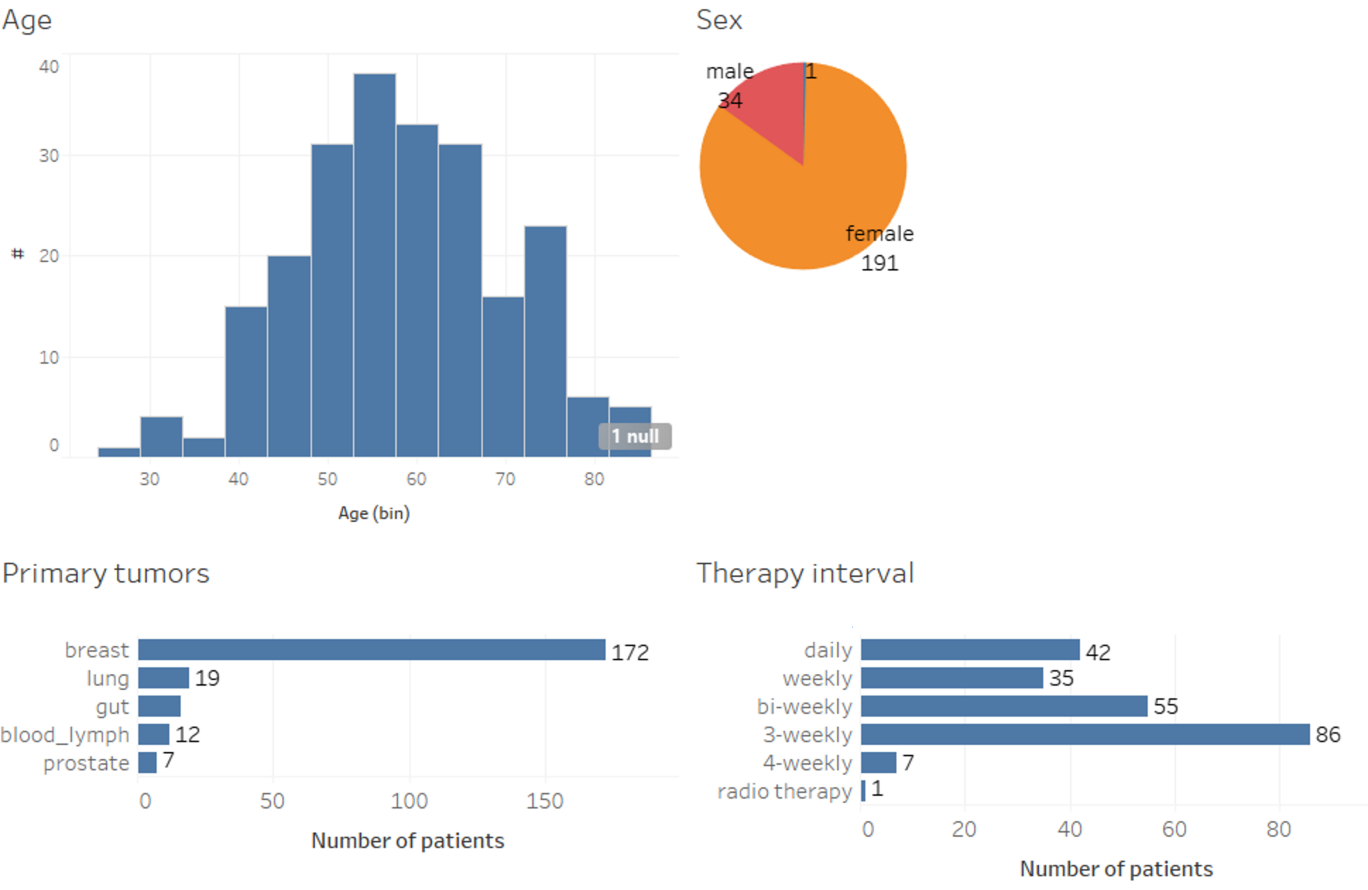
Patient demographics.

Figure 1 shows the demographics of the 214 patients included in this study in terms of age, sex, their primary tumor, and systemic therapy. Patients aged 18 or older with breast, colon, prostate, and lung cancer, as well as hematological malignancies, initiating adjuvant or neoadjuvant systemic therapy were eligible to participate after giving written informed consent. The mean age of patients in this cohort was 58.4. In addition, participants had to speak German and own a smartphone. Eligible participants in the observational study had been recruited consecutively and without pre-selection.

Recording of well-being and symptoms usually started on the day of the initiation or change of anticancer treatment and continued during an observational period of 12 weeks. Patients were assigned to medical oncology visits every 3 weeks for shared reporting. During consultation visits, doctors and nurses reminded the participants to use the app, and participants additionally received push notifications every 3 days.

Furthermore, the app randomly selected, at regular intervals, two patient-reported symptoms that had been recorded during the past 20 days; patients and doctors were then prompted to perform a detailed and shared review of these symptoms in order to focus on the collection and appropriate interpretation regarding awareness and guidance for symptom severity grading (11, 12).

Less than one third (*n* = 51; 28%) of the patients received treatment for advanced disease with non-curative intention, and 17 different chemotherapy regimens were administered (not shown), including anti-hormones, CDK4/6-inhibitors and immunotherapies. For the data analysis, informed consent was obtained from all patients.

**Figure 2:**
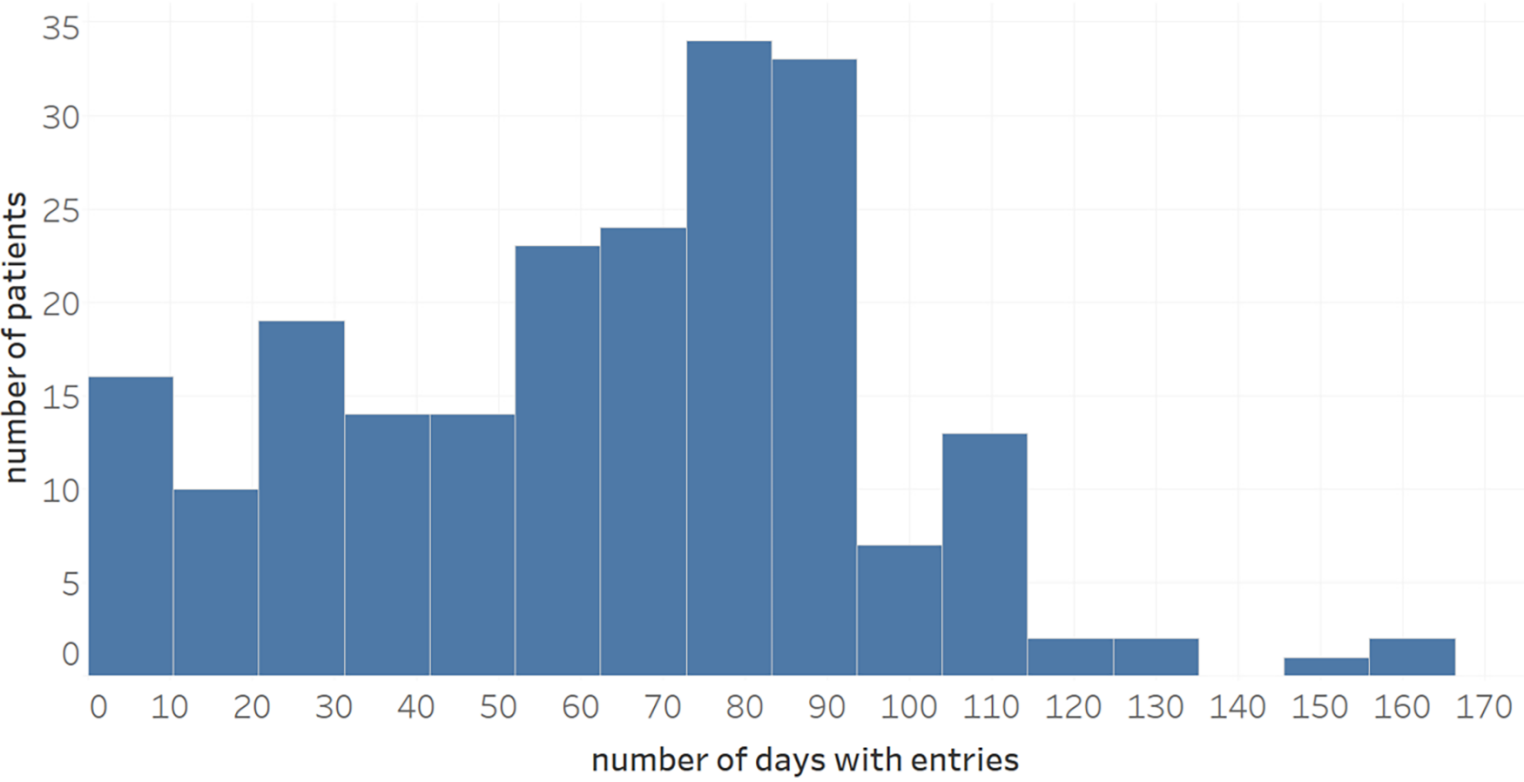
Distribution of number of diary entries during a maximum observational period of 170 days.

Overall, the dataset available for analysis contained 16,670 diary entries where a diary entry refers to an entire day's worth of patient entries. Figure 2 shows the distribution of the number of symptom entries per patient (mean: 63.0). When not aggregated day-wise, the data contains 76,385 individual entries of symptoms, wellbeing, use of medication or notes. Regarding data accuracy and the use for subsequent ML analysis, a total of 181 patients had performed at least one intended symptom review with their physician, and, from a subset of 110 patients (60.8%), more than two collaborative symptom reviews of patients with their physicians were available for analysis, indicating a presumably high data quality (11, 12).

### Unplanned visits

Within the overall observational period, 40 patients had experienced unplanned visits, which amounted to 54 events overall, including 31 unplanned medical consultations and 23 hospitalizations. No other serious adverse events (SAEs) were recorded during the entire study period. We had expected a higher rate of unplanned consultations and hospitalizations—which would presumably have led to an increased quality of ML and prediction; however, the retrospective nature of the study did not allow for any expansion of data available for analysis.

### Data characteristics and preparation

Apart from patient demographics and unplanned visits, our dataset also contained diagnosis details (as free text), as well as the ePROs symptom entries, well-being, medication, and additional free-text notes. Table 1 shows an overview of all features that were available.

**Table 1:** Data available for patients' diary entries (ePROs).

Attribute(s)	Number of attributes	Description	Type/values
Birth year	1		Numeric
Sex	1		{Male, female}
Primary tumor	1		{Breast, gut, blood/lymph, lung, prostate}
Wellbeing	1	Subjective wellbeing	[0…100]
Therapy form	1	Frequency of treatment	{Daily, weekly, bi-weekly, 3- weekly, 4-weekly}
Drugs	88	Cancer drugs, other drugs	[1,nan]
Symptom grading	52	Strength of relevant symptoms, based on CTCAE	[0…1,nan]
Diagnosis terms	246	Terms occurring in diagnosis details of patient	Numeric (TF/IDF)
Note terms	311	Terms occurring in patient notes	Numeric (TF/IDF)
Unplanned visit	1	Class attribute	

In terms of data missingness [see reporting guidelines for ML studies in (22)], the demographic attributes (age, sex and primary tumor) were almost complete, with only age and sex missing for one out of the 214 patients. Values for diagnosis and therapy form were also available for all patients. A value for the patient's wellbeing is missing in 931 of the 16,670 entries and only 5,000 of these entries come with a note captured by the patient. Finally, since patients can capture multiple symptoms and drugs per day, we pivoted these, i.e., created columns for each symptom and drug, where the maximum symptom strength reported on a day was used in case of multiple entries for the same symptom. This pivoting operation results in a very sparse matrix where some symptoms only have a few non-null values. Therefore, we needed to make sure that our chosen algorithm is able to deal well with missing values.

Each patient-day combination in the dataset available for analysis represented one training example where we assigned a class attribute called “unplanned visit” to each instance as follows: we labeled the attribute as “yes” not only on the day when an unplanned visit had occurred, but also on the three preceding days. This approach aligned with our goal of creating an early warning system (EWS) that anticipates issues several days in advance. This allows to raise timely warnings for potential problems occurring on weekends (e.g., predicting a problem that will occur on Sunday evening is predicted already on Friday morning; as such, a visit during regular practice hours would become feasible). Another positive effect of using this rather large time frame is that the number of positive training examples (i.e., flagged diary entries) increases when compared to a shorter timeframe of e.g., 48 h. Finally, this approach resulted in 166 diary entries being flagged as “yes”, i.e., as ones where an alert should have been raised. This corresponded to roughly 1% of all entries, indicating a heavily imbalanced data set where the number of examples for one outcome is much larger than the number of examples for the other outcome (here: non-critical vs. critical situations), a more challenging prediction of the rarer outcome was expected.

As mentioned above, drugs and symptom gradings were represented using a set of attributes where each attribute indicated the presence or grading of the symptom on a specific day. To encode symptom grading on a scale from 0 to 100, patients were provided with a guideline that included definitions and descriptions of symptoms and their associated grading according to the CTCAE. Symptoms that had *not* been reported by a patient on any given day were encoded as “null”/”nan” values, which were ignored when constructing rules. This was found to be more appropriate than encoding them with a value of 0 as the latter approach led to the identification of a large set of non-relevant rules with “negative conditions” and consequences—e.g., “if patient has no fever and no cough, then do not raise an alarm”. We also converted the free-text attributes “diagnosis” and “notes” using a so-called bag-of-words approach with TF/IDF weights [term frequency/inverse document frequency, see (23), with the resulting attributes prefixed as “diag” and “note”.

We deliberately prioritized readability over predictive performance, avoiding approaches like “word embedding”, to maximize rule interpretability for medical experts. No further pre-processing was applied to the data.

### Human baseline

Before applying machine learning to our data, we wanted to explore the ability of human experts to predict adverse events. We asked a human expert to define a set of conditions under which he would expect such events to occur. The expert first defined problem categories, i.e., areas that could lead to complications in cancer patients under treatment, including infections, kidney-related, lung-related, as well as cardiac/respiratory problems and side effects of cancer drugs. For these categories, the expert then defined more concrete conditions that read as follows:

• Fever, fatigue, joint pain and cough (infection)
• Strong diarrhea (infection)
• Burning during urination (infection)
• Weight gain and shortness of breath (kidney)
• Nikotin abuse (stated in diagnosis) and chest pain (lung)
• Weight gain, fatigue and either shortness of breath or chest pain (cardiac/respiratory)
• Loss of appetite, fatigue and weight loss (side effects of cancer drugs)

We then used the CTCAE scales to define suitable thresholds of symptom strengths for the symptoms occurring in these rules and applied the rules to the data.

It turned out that the rules did not discover a single adverse event recorded in the data, i.e., we start with a system that achieves 0% precision and recall. It is important to note here that this does not lead us to believe that the human knowledge is worthless or not applicable at all. Rather, it seems necessary to complement it with more detailed insights from the actual patient data—e.g., to find better thresholds—in order to increase the recall.

### ML approach

It has been shown by (20) that it is possible to derive rules for the prediction of medical conditions using machine learning. In (20), it is further shown that an inspection and rating of ML-discovered rules by humans can lead to the detection of hidden confounders and poorly generalizing rules. Interestingly, human intervention in (20) did not improve the predictions on the test set—but it did so on another data sample, i.e., in an out-of-sample evaluation. This is an important finding: while humans are often limited in their ability to formulate precise rules (see previous section), they are in fact capable of spotting conditions in ML-generated rules that do not generalize well: if the data used for training an ML system has weaknesses, such as confounders or spurious correlations, humans can use their general medical knowledge to spot and remove them.

The overarching goal of our approach is thus to empower medical experts to design a rule-based early warning system (EWS) with the help of machine learning.

Because of the given imbalance of the data (only 1% of entries being flagged as alert-worthy) and the somewhat limited amount of our data, we did not expect a machine-learned rule set to exhibit a good performance. Instead, we used ML mainly as an aid and inspiration for the human expert—to suggest rules that are based on real occurrences of adverse events and that the expert might not have readily thought of. This approach is based on the assumption that humans and ML algorithms both have strengths that can complement each other, where human intervention can be especially useful e.g., in cases where one tries to predict rare events (24).

To this end, we made the ML algorithm sensitive to the few adverse events (see description of cost-sensitive classification below), accepting a rather low precision. At the same time, we enforced a certain coverage (support) of rules by applying a weight threshold to rule conditions. This approach results in rules that are rather general—but because of the increased sensitivity, some of them might still be too specific. Finally, we expected that both a lack of precision and an over-specialization of rules can be corrected by humans.

Therefore the EWS consisting of a set of rules was constructed as follows:

- We applied the simple, but often quite effective, rule-learning algorithm RIPPER (25) to train an ML model on the data described in the previous subsection. The resulting model consisted of rules that had certain combinations of symptom strengths, diagnosis terms, etc., as condition and—when the conditions were fulfilled—would predict the occurrence of an unplanned visit. Our main rationale for choosing a rule learner was the fact that rules are very easy to interpret by any human due to their intuitive if-then structure (18). On the other hand, they stand out among other interpretable models in that it is rather easy to *modify* them, e.g., by adding condition clauses. The effect of such a modification (namely increasing the specificity of the rule/decreasing its coverage) is also obvious because rule conditions are expressed as a conjunction of clauses.
- The rule learner was combined with cost-sensitive classification (26) with various cost matrices. In our reported results, false positive predictions (”false alarms’”) were assigned a cost of 1, while false negatives (undetected critical situations) had a cost of 10. Our medical experts endorsed this approach, accepting a higher number of false alarms to identify additional critical situations, i.e., reaching higher sensitivity, at the cost of lower specificity. Interestingly, when using a 1:1 cost ratio, *no* rules were generated. Using a 1:20 ratio instead of 1:10 yielded similar numbers and characteristics of rules.
- To avoid overfitting of the algorithm to certain specifics of patients or situations in the training data, we complemented the increased sensitivity with a minimum weight threshold for the conditions of rules. This was done in such a way as to allow only rules that cover at least 5 positive examples. More precisely, the parameter determining the minimum total weight of instances in a rule was set to 42. Since each unplanned visit will generate up to 4 positive examples (the day of the visit and the three days before), and since the weight of each flagged entry is slightly above 9, this ensures that a rule usually covers at least two distinct critical situations that resulted in a visit. We will see later that the visits discovered by our rules also stem from at least two different patients in each case. Finally, we set the number of folds to three such that two folds were used for growing rules and one for pruning them.
- Rules were ranked by their cost and then presented to medical experts, together with some performance metrics on the entire training data (above all precision and recall). Two oncological experts (A.T., M.M.), each with more than 20 years of experience in internal medicine and medical oncology, examined the ML rules in ranked order and decided whether to accept, remove or modify a rule, e.g., by removing or adding conditions. These modified rules underwent statistical evaluation and were accepted if their cost on the test set was deemed acceptable.

## Results

### Performance of ML models

In order to evaluate the model's performance, we constructed a training set covering 85% of all our entries and used the remaining 15% of the data (corresponding to 2,500 entries) as a test set. To avoid leakage of patient-specific data from the training into the test set, we made sure that patients whose data were used in the training set did not appear in the test set.

As expected—given by our approach of assigning a high cost to false negatives—there was a substantial amount of false alarms on the test set (122). The machine-learned rule set achieved a recall of 19%, successfully identifying 6 out of the 32 critical situations in the test set. Because of the 122 false alarms, the precision of this rule set was rather low at about 5% (while keeping in mind that for 2,346 entries no alarm was raised correctly). All statistics and metrics were collected using the WEKA machine learning workbench (27) and the corresponding evaluation methods had been applied according to state-of-the-art ML algorithms and data pre-processing tools (28).

Assuming—as introduced above—a cost of 1 for false alarms and a cost of 10 for missed critical situations, the baseline of never raising an alarm causes costs of 320 (all 32 situations are missed, each at a cost of 10), whereas our rule set causes slightly higher costs at 122 (false alarms) + 260 (missed alarms) = 382. If we assume higher costs for missed alarms, we will observe that our initial rule set causes lower costs than the baseline. Doing so may be justified in practice (and note that the costs used for evaluation do not need to be the same as the ones for cost-sensitive learning!) when considering that a missed alarm can be life-threatening in some cases. For instance, a ratio of 1:20 has been assumed for a fall prediction model in (29) emphasizing, however, the difficulty to estimate these costs precisely, which also applies in our case.

In any case however, without human intervention, these results may not be very useful, although they may improve with a larger dataset. Thus, we would like to re-emphasize that the purpose of applying ML in our approach is mainly to aid humans in setting up a rule base. Humans may change rule conditions (e.g., inserting an additional clause) and decide on the rule consequence, which is not necessarily to contact a physician. For instance, the precision of many rules might be improved by triggering the capturing of additional (vital) parameters, e.g., measuring blood pressure or making blood tests.

### Rule interpretation and modification

**Figure 3:**
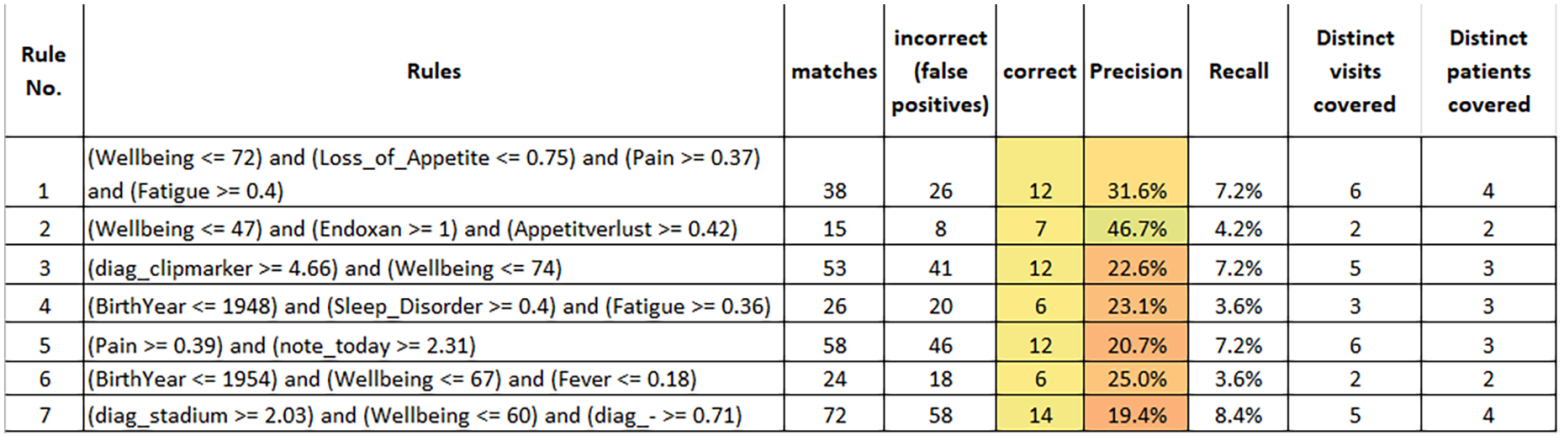
The seven rules with lowest cost discovered by our cost-sensitive rule learner.

All in all, our configuration resulted in 7 rules which are summarized in Figure 3. These results were obtained by training rules on the entire dataset and applying them accordingly. The reported figures also apply to the entire dataset. The first observation is—as already mentioned—that all rules cover 2 or more distinct patients. Taken together, the rules cover 56 of the 166 flagged entries in the entire dataset.

The inspection of the rules by medical experts with long standing clinical experience in internal medicine, medical oncology, and study conduct, provided us with the following insights:

- It was easy for the medical experts to interpret the meaning of the rules. This is the biggest strength of rule learners, also implying the possibility to adjust the rules (see below). Although theoretically the order of rules needs to be considered when interpreting the output of RIPPER, this is not the case for a binary classification problem like ours such that experts can understand and judge rules independently.
- All rules except the last rule were considered both reasonable and useful. In the following, we only discuss about rules 1–6.
- Some of the rules were considered slightly too general, e.g., rule 5 and 6. However, for all rules involving pain and fever (i.e., rules 1, 5 and 6), the experts judged that additional data capture could be useful to increase their precision, e.g., by performing a CRP test to identify potential infections. If we take rule 6 as an example, the medical experts judged that the low level of fever and the somewhat reduced wellbeing did not *per se* justify a warning being raised. They assumed that the patient (who had come for an unplanned visit) was a rather careful person and that, with a high probability, the situation would have improved without intervention. However, applying a CRP test to verify this assumption would have been informative and useful. Another example rule that was finally discarded (not shown in the figure) stated that a warning should be raised when the diagnosis of a patient contained “metastasized carcinoma” and abdominal pain was indicated above 0.42. Here, the experts judged that the rule condition was too generic since the abdominal pain could have many causes (e.g., constipation or aszites…). In such cases, their recommendation was to monitor the symptom trend and react when the pain level increases (i.e., use a somewhat higher threshold in the rule). In summary, the experts identified some rules that they found slightly too weak and where they would only raise a warning after doing additional tests (e.g., CRP) or after further monitoring the situation.
- For rule 3 (patients with clipmarkers and reduced wellbeing), the experts suggested adding an extra condition of either a raised level of nausea, fatigue or pain to make the rule more precise.
- Rule 2 suggests raising a warning in case of a patient receiving treatment with the medication Endoxan® (Cyclophosphamide), reporting a rather strong loss of appetite and well-being dropping below 47. This likely indicates a situation of systemic chemotherapy where intensified measures for the alleviation of treatment-associated symptoms could be suggested. Thus, learned rules may lead to different kinds of alternative actions, implying some sort of “level-setting procedure” of the early warning system, where obviously not all raised warnings must result in a patient’s hospitalization.
- We could observe that the learned rules—and this is also true for the rules that are not shown here—make use of most of the attribute types that were used as an input, i.e., age (rules 4 and 6), wellbeing (rules 1–3 and 6), drugs (rule 2), symptoms (all except rule 6), diagnosis terms (rule 3) and patient notes (rule 5). In general, the attributes sex, primary tumor and therapy form did not appear frequently in rules. We did not quantify the contributions of attributes here in more depth, since this seems less adequate for rule learners and might be misleading.
- We are aware that different treatments might require monitoring of various parameters. The medical device app “medidux^TM^” was not designed for sending out questionnaires according to the type of cancer or related treatment, but rather offers the opportunity to choose from more than 93 different symptoms that can be reported dynamically and thus will presumably cover all relevant side effects, as has been indicated in previous work (11, 12). On average, more than 3 different symptoms were entered per patient and day. However, most of the rules are derived from symptoms reported by breast cancer patients, which might be considered a limitation of this study.

While fewer than 18.7% (40/214) of the participants treated for solid cancer (breast, colon, lung, prostate) and hematologic malignancies required unplanned consultations or emergency services due to treatment-related side effects and toxicities, our analysis shows a potential to develop an early warning system jointly between an ML algorithm and human experts. Although the limitations of the data imply that learned rules have a low precision, we saw several examples of rules where experts were able to suggest measures for increasing precision, e.g., by performing additional tests or by inserting additional conditions.

Importantly, no serious adverse events related to the use of the app were recorded during the entire observational study period.

## Discussion and conclusions

In cancer outpatients, only limited data exist on how to provide algorithms for improved real-time monitoring and intervention to reduce symptom severity and to predict situations where unplanned visits will become likely (13, 21). Here, we propose two approaches for high-rate EWS computation and time-series prediction based on standardized and dynamically reported symptoms and vital parameters indicated by cancer patients who experienced unplanned consultations and hospital admissions.

Our data indicate that machine-learned rules—when applying a cost-sensitive classification to make them more sensitive—could detect a certain number of critical situations, respectively novel alert conditions, in the cohort of outpatients. In their original form, these rules did not allow for reliable predictions on a test set. However, according to our analysis, medical experts can validate these proposed rules and suggesting modifications to specific rule conditions or alternative types of alerts to be raised based on longstanding clinical and oncological experience. While most of these suggested modifications can only be evaluated in future work, one referred to an additional clause in the rule condition. Evaluating it on our data revealed that—as expected—it increases the precision of the rule, while also decreasing its recall.

Overall, the most valuable contribution of human-machine collaboration to the EWS is certainly the suggestion of (alternative) actions to be performed when a rule fires. Because alarms are relatively rare, it would be virtually impossible to learn these actions from the data only. However, since the medical experts were able to understand the rules, it was not difficult for them to suggest a suitable course of action to be undertaken.

In fact, this is the underlying idea for the procedure of interactive ML (30) as presented here: on the one hand, the discovered rules indicate that ML is likely to identify patterns in electronically captured PROs that medical experts would not think of readily. On the other hand, ML alone using cost-sensitive classification renders newly discovered rules sensitive, but, at times, imprecise. Cost-sensitive learning was, as discussed above, a necessity because of the highly imbalanced data (1% critical situations), which would otherwise lead to trivial rules that never raise any alert—whereas other authors have used e.g., oversampling to increase the sensitivity of their alert systems (31).

The imprecision of the discovered rules, however, can potentially be compensated by the experience of medical experts who can estimate whether a rule may generalize beyond the specific training data or not and thus help to increase precision even in the face of comparatively little training data (20). Thus, a human-machine collaboration might successively facilitate an ever more reliable and hybrid rule-based EWS, as experts interact with the ML model.

While medical experts appear confident in understanding and creating reverse interpretations of the rules, iteratively modified rules can be expected to generalize better to unseen data because the modifications usually eliminate case-specific aspects that may not apply to most other patients (20). Moreover, thorough medical expert review would allow to define a variety of rule consequences, implementing plausible and more diverse recommendations to patients and caregivers that would circumvent unplanned physician consultations or admission to hospital.

Of note, when comparing the effectiveness of the derived rules to the rules that our medical experts had formulated without the support of ML in advance, our results offer a clear improvement over the human baseline and justify the use of machine learning as an “inspirational” tool for medical experts.

The present study was limited with respect to the patient demographics (and absolute numbers of the data available for analysis, i.e., bias through predominantly female patients with breast cancer treatments), the retrospective character of the ML, and the limited number of medical experts for review and adaptive rule management. It is worth noting here that none of the discovered rules used the attribute “PrimaryTumor”, i.e., the cancer type. This indicates that the patterns underlying the recorded adverse events are of a general nature, not specific to any particular cancer type.

To further validate this insight, we ran an additional experiment where we filtered the data so that only entries of breast cancer patients were retained, thus reducing the size of the dataset by 15%, from 16,670 diary entries to 14,060. We observed that the resulting rules had a small overlap with the ones learned from the full dataset and that the rules that differed from the initial ones were harder to interpret. Taken together, these additional findings suggest that predictive performance is mostly influenced by the size of the training data and does not strongly depend on the purity of the data with respect to the cancer type of involved patients. Thus, future work should focus on the collection of larger sets of training data.

Finally, to perform a real-time EWS and initiate a prospective confirmation study, further software development and the proper calculation of the needed number of patients and events would be required. We also did not aim at discovering SAEs for patients as early as possible by using multi-modal data, since the retrospective analysis did not support an extension of these events. However, we believe that next generation medical device apps and their interoperability with IoT devices and diagnostic laboratories might boost the design and improvement of predictive approaches. In fact, we saw that our medical experts advocated the use of such devices to confirm the alerts raised by certain rules (mostly the ones incorporating pain and fever) and thus increase the precision of these rules. In addition, a more precise prediction will likely be achieved with more and continuous data samples, which could result from wearables and related sensors.

Besides the extended recording of standardized and structured ePROs, interactive ML systems for patient centered health care will benefit from more plentiful data, by integrating dynamic measurements of vital parameters, real-time laboratory tests and cognitive abilities that will allow to predict critical situations far more accurately and timely (13, 15, 21). Finally, with dedicated medical experts validating novel sets of rules, the proposed system can be extended continuously as new ePRO data become available and may conceivably outperform personnel-intensive monitoring of cancer outpatients.

## Data Availability

The raw data supporting the conclusions of this article will be made available by the authors, without undue reservation.
